# False-Negative Platelet Factor 4 Antibodies and Serotonin Release Assay and the Utility of Repeat Testing in the Diagnosis of Heparin-Induced Thrombocytopenia and Thrombosis

**DOI:** 10.1155/2019/1585014

**Published:** 2019-01-08

**Authors:** Tarig Omer, Naresh Mullaguri, Pravin George, Christopher R. Newey

**Affiliations:** Department of Neurointensive Care, Cerebrovascular Center, Cleveland Clinic Foundation, 9500 Euclid Avenue, Cleveland, Ohio 44195, USA

## Abstract

**Objective:**

To report a case of false-negative serological tests in the diagnosis of heparin-induced thrombocytopenia (HIT) followed by a brief review of the literature on this topic.

**Case Presentation:**

A 75-year-old Caucasian female patient was admitted with a traumatic right ankle fracture that required open reduction and internal fixation. Despite postoperative subcutaneous heparin chemoprophylaxis, she developed deep vein thrombosis (DVT) and pulmonary embolism (PE) on day 4 and subsequently started on continuous heparin infusion. On day 5, she suffered a stroke from a complete occlusion of the right common carotid artery with tandem occlusion of the right middle cerebral artery. She underwent successful thrombectomy of both arteries. The proposed stroke mechanism was paradoxical embolism through a patent foramen ovale. Over the next few days, thrombocytopenia was noted, the heparin drip was stopped, and HIT antibodies (antibodies targeting the complex of platelet factor 4 and heparin; PF4-H AB) and serotonin release assay (SRA) tests were sent. Because of the suspicion for HIT, she was started on bivalirudin with subsequent improvement in platelet count. Initial PF4-H AB and SRA tests were negative, bivalirudin was stopped, and heparin was restarted. Subsequently, her platelets trended down, again raising clinical suspicion of HIT. Repeat PF4-H AB and SRA testing resulted positive.

**Conclusions:**

A positive SRA in the appropriate context is considered for the diagnosis of heparin-induced thrombocytopenia. This case report highlights that false-negative serological evaluation is possible early in the course of the disease. Repeat testing is recommended in patients with high clinical suspicion.

## 1. Background

Heparin-induced thrombocytopenia (HIT) should be suspected in patients with sudden thrombocytopenia and signs of thrombosis after exposure to unfractionated or low-molecular weight heparin [1, 2]. The pathophysiology of HIT involves endogenous antibodies targeting platelet factor 4 complexed with heparin (PF4-H AB) that causes catastrophic platelet aggregation resulting in predominantly venous and rarely arterial thrombosis and embolism [3]. HIT is considered a rare etiology for ischemic stroke outside of the context of cerebral venous thrombosis [4, 5]. Once HIT is suspected, systemic hypercoagulability should be managed with nonheparin anticoagulants such as bivalirudin or argatroban. Serological evaluation of PF4-H AB antibodies and SRA should be completed before restarting a heparin anticoagulant [1, 3]. The PF4-H AB antibody is considered a very sensitive screening test, and together with a positive SRA in the correct clinical context, it is considered to be virtually diagnostic of heparin-induced thrombocytopenia [6–8]. False-negative PF4-H AB and SRA are reported only early in the course of disease and in patients receiving immunosuppression, massive blood transfusions, or plasmapheresis [8–12]. This manuscript highlights false-negative early serological testing in HIT, stresses the importance of repeat serological testing if there is strong clinical suspicion, and provides a brief review of the pertinent literature.

## 2. Case Presentation

**Figure 1:**
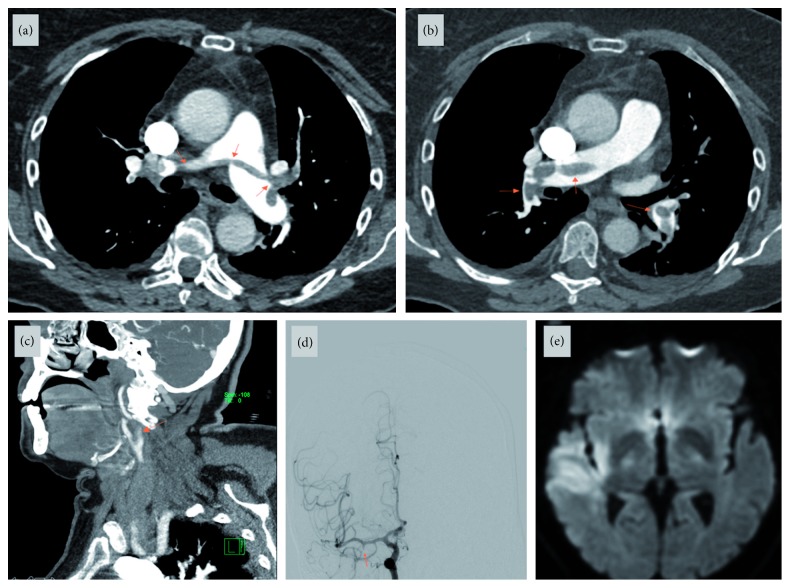
(a, b) Computerized tomography angiography of the chest (axial images) showing “saddle” shaped thrombus in the pulmonary artery bifurcation extending into both trunks (arrows); (c) computerized tomography angiography of the neck (sagittal view) showing right common carotid artery bifurcation showing “saddle-shaped” thrombus in the external and internal carotid arteries (arrow); (d) digital subtraction angiography with selective right internal carotid artery injection showing middle cerebral artery inferior division occlusion (arrow); (e) magnetic resonance imaging of the brain diffusion-weighted imaging axial section showing right frontotemporal area infarction corresponding to the middle cerebral artery inferior branch occlusion.

**Figure 2:**
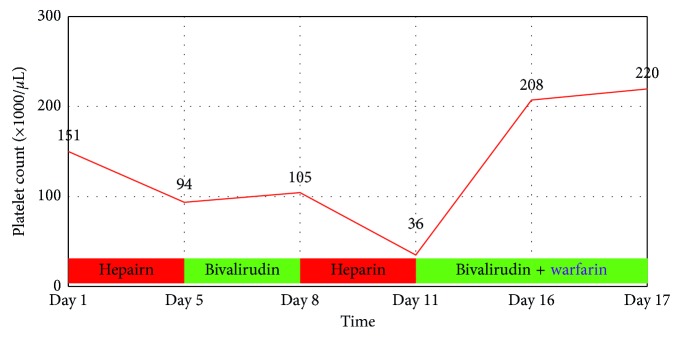
Patient's platelet trend during hospitalization while on unfractionated heparin and bivalirudin infusion.

A 75-year-old Caucasian female with a history of hypertension and well-controlled type 2 diabetes mellitus was admitted after open reduction and internal fixation of an ankle fracture. She was treated with subcutaneous unfractionated heparin (5000 IU three times a day) for deep vein thrombosis (DVT) prophylaxis from day 1. On day 4, a DVT and pulmonary embolism (PE) workup was completed after she developed acute shortness of breath and tachycardia with hypoxemia. DVT and subocclusive saddle-shaped PE were both noted (Figure 1), and she was started on a continuous heparin infusion. The following day she developed sudden onset of left-sided weakness and rightward gaze deviation concerning acute ischemic stroke. CT angiogram of the head and neck and subsequent MRI brain revealed a stroke caused by right-sided common carotid artery occlusion with thrombus extending into the external and internal carotid arteries. A tandem occlusion of inferior branch of the right middle cerebral artery was also noted on cerebral angiography (Figure 1). The patient underwent successful mechanical thrombectomy of the right carotid artery bifurcation thrombus. After the endovascular procedure, thrombocytopenia (153,000/*µ*L to 94,000/*µ*L) was noted. Clinical suspicion was raised for HIT and PF4-H AB, and SRA tests were sent. Heparin infusion was stopped, and she was started on continuous bivalirudin infusion. Transthoracic echocardiogram with bubble study revealed a patent foramen ovale (PFO) with right to left shunting, and the etiology of stroke was presumed a paradoxical embolism. On hospital day 7, PF4-H AB (optical density (OD) < 0.103) and SRA tests resulted negative with uptrending platelet count (105,000/*µ*L) (Figure 2). Bivalirudin was subsequently stopped, and the patient was restarted on therapeutic continuous heparin infusion due to reduced suspicion of HIT as a result of the negative serological tests. From day 8, platelets started to downtrend to 36,000/*µ*L. Clinical re-emergence of HIT was suspected, and heparin infusion was held. Repeat PF4-H AB antibody and SRA tests were sent and resulted positive. The patient was treated with bivalirudin and bridged successfully to warfarin with recovery of platelet count (205,000/*µ*L). She suffered no further thromboembolic events and was eventually discharged to an inpatient rehabilitation facility.

## 3. Discussion

PF4-H AB and SRA testing can be reported as falsely negative early in the course of the disease in patients with HIT possibly due to low antibody titers [11, 12]. In cases with high clinical suspicion, repeat testing is warranted [8, 12]. DVTs and PE are the most frequent complications of HIT, followed by peripheral arterial thrombosis and stroke [3]. Myocardial infarction is uncommon [13]. The typical onset of thrombocytopenia in HIT occurs 5 to 10 days after the initiation of any heparin therapy [3]. HIT within 24 hours of heparin exposure may be suspected if the patient was exposed to heparin in the previous one to three months and has circulating antibodies [3, 14]. Delayed-onset HIT, in which thrombocytopenia and thrombosis occur after heparin has been withdrawn, is also described; however, the incidence is unknown [14, 15]. Life-threatening thrombosis, amputation, or death is reported if the diagnosis of HIT is missed, characterizing the severity of the disease [1, 3].

There are two types of immunoassays to detect HIT. The first type is an enzyme-linked immunosorbent assay (ELISA) to measure PF4-H AB antibodies quantitatively. The second type is a functional platelet assay called the SRA, which measures the ability of the antibodies from a patient's serum to activate platelets. The PF4-H AB ELISA test is widely available, fast, and straightforward to interpret but has a high incidence of false-positive results [16]. The SRA strongly correlates with the presence of the HIT syndrome and is considered the gold standard with sensitivity and specificity of >95 percent; however, generally it takes a longer time to result and is not widely available [1, 3, 7]. The SRA is generally reserved for those with indeterminate immunoassays (i.e., ELISA with optical density (OD) from 0.40 to 2.00) or those for whom the clinical picture and immunoassay results are discordant [6]. Cases of false-positive and false-negative PF4-H AB ELISA with SRA test have been reported [9–11]. Case reports of the negative serological evaluation of HIT are also described by Jones et al., Patel and Varga early in the disease course [11, 12]. Senzel and Coldren reported false-negative assay after massive blood transfusion, possibly due to dilution of antibodies below the limit of detection [10]. Iluonakhamhe et al. report a case of HIT in the setting of acute intracerebral hemorrhage managed with plasmapheresis. After four cycles, both heparin antibody ELISA and functional assays became negative [17].

In our patient, the false-negative PF4-H AB and SRA may have been secondary to low levels of antibodies early in the disease [11]. It is also possible that there was a lab technical error. It is suggested and highlighted by this report that clinicians be aware of false-negative results when testing for serological markers of HIT. In patients with high clinical suspicion for HIT, even in the presence of negative serological testing, we propose that clinicians closely follow their clinical course and platelet trends and switch to a nonheparin anticoagulant as clinically indicated. Repeat PF4-H AB antibody and SRA testing may help confirm a HIT diagnosis if initial early test results are negative.

## 4. Conclusions

PF4-H AB ELISA with SRA testing in the appropriate clinical context is considered the gold standard for the diagnosis of HIT; however, false-negative serological evaluation is possible early in the course of the disease. Repeat testing of PF4-H AB ELISA and SRA is recommended in patients with high clinical suspicion who initially test negative.
